# Severe psoriasis vulgaris complicating pemphigus vulgaris: A case report

**DOI:** 10.1097/MD.0000000000042354

**Published:** 2025-04-25

**Authors:** Lanying Wang, Guixian Guo, Shanshan Tang, Shaoqin Sun, Ran Wu

**Affiliations:** a Guizhou University of Traditional Chinese Medicine, Guiyang, China; b Medical Department, The First Affiliated Hospital of Guizhou University of Traditional Chinese Medicine, Guiyang, China; c Department of Dermatology, The First Affiliated Hospital of Guizhou University of Traditional Chinese Medicine, Guiyang, China.

**Keywords:** case report, methotrexate, nail psoriasis, pemphigus vulgaris, psoriasis vulgaris, secukinumab

## Abstract

**Rationale:**

Currently, the main comorbidities of psoriasis are cardiovascular, metabolic, liver and kidney, autoimmune, and psychological disorders. Psoriasis associated with pemphigus is relatively rare. This rare disease presents with significant clinical, diagnostic, and therapeutic challenges.

**Patient concerns:**

The patient was a 71-year-old man with recurrent erythema, papules, and scales with itching all over the body for 11 years, accompanied by blisters and erosions for more than 1 month, and aggravated by generalized eruption for 1 week. The patient was diagnosed with pemphigus vulgaris and admitted to our hospital with intravenous methylprednisolone combined with a conventional oral antihistamine and topical hormonal ointment. The patient’s symptoms significantly disappeared. The patient complained that his condition recurred easily after discontinuing medication, which seriously affected his daily life and sleep. The patient also had essential hypertension, nail psoriasis, tinea pedis, and onychomycosis.

**Diagnoses:**

The patient was diagnosed with psoriasis vulgaris with coexisting pemphigus vulgaris.

**Interventions:**

The patient was treated with modest doses of glucocorticoids combined with secukinumab and methotrexate.

**Outcomes:**

The patient’s generalized skin lesions and fingernails and toenails of the hands and feet healed virtually. There was no recurrence at 8 months follow-up, and no adverse effects were noted.

**Lessons:**

Moderate-dose glucocorticoids combined with secukinumab and methotrexate may be an option for treating psoriasis and pemphigus vulgaris. This case allows us to consider whether we can treat psoriasis vulgaris combined with pemphigus vulgaris based on a common pathogenesis, and to guide us in clinical dosing.

## 1. Introduction

Psoriasis is a common immune-related chronic relapsing inflammatory skin disease. Pemphigus is a group of autoimmune herpetic dermatoses caused by breakdown of intraepidermal acanthoses. Here, we report a case of severe psoriasis vulgaris complicated by pemphigus vulgaris (PV). The patient also had essential hypertension, nail psoriasis, tinea pedis, and onychomycosis. In our case, the initial treatment was intravenous methylprednisolone, followed by oral prednisone acetate. Owing to the poor control of the patient’s condition, moderate-dose glucocorticoids combined with secukinumab and methotrexate were administered, and the patient’s generalized lesions, fingernails, and toenails were essentially normalized. In addition, we briefly discuss the relationship between psoriasis and pemphigus.

## 2. Case report

**Figure 1.**
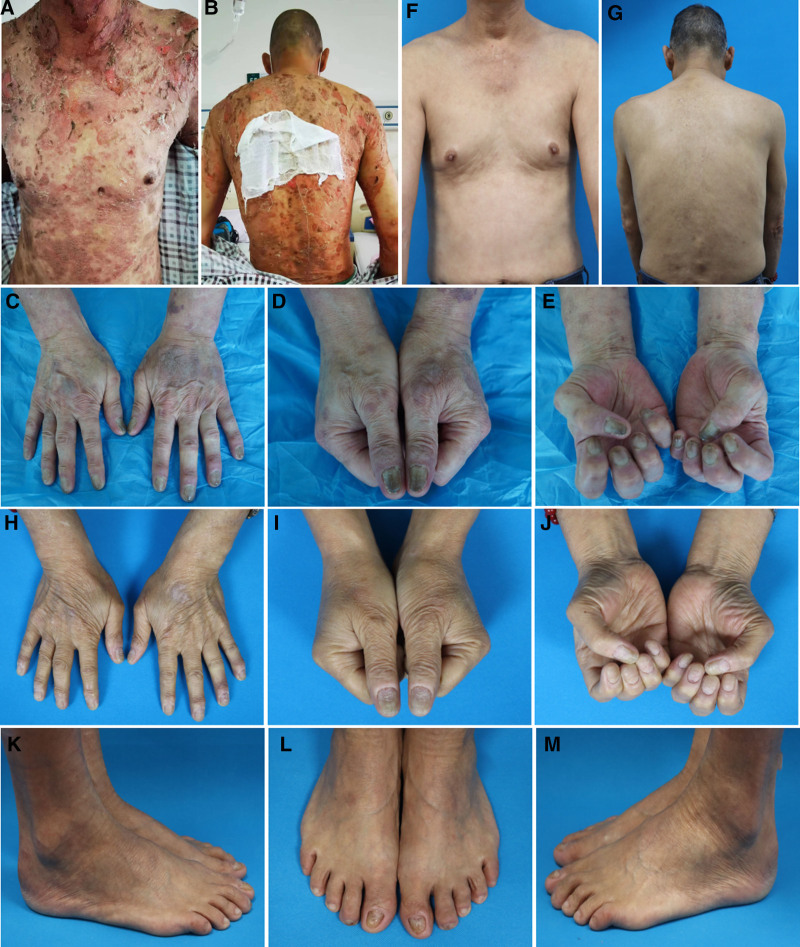
(A–E) The entire body is scattered with flaky erythema of various sizes. Based on erythema, flaccid blisters and blisters can rupture, forming a large reddish erosion, small amount of exudation, and part of the crusts with dry scales. The surface of the nail plate of both hands was uneven, cloudy, hypertrophic, and lacked a luster. Some nails showed gray-black and pitting-furrowed changes with defects at the distal nail edge. (F–M) No psoriasis or pemphigus lesions are observed on the skin of the entire body, and the finger (toe) nails of the hands and feet are largely normalized.

A 71-year-old male patient with an 11-year history of plaque-type psoriasis visited our hospital on October 01, 2022, complaining of scattered erythematous patches and papules on the abdomen without obvious triggers 1 month prior, along with new blisters and vesicles, which were slightly relieved by self-administered topical ointment treatment. However, 1 week prior, the lesions gradually spread to the whole body and appeared diffuse, with various sizes of flaky erythema, which can be seen on the flaccid blisters, blister rupture, the formation of a large reddish erosion, a small amount of exudation, part of the crusts, with dry scales, with itching of the area of skin rash, pain, and Nikolsky sign was positive. The surface of the nail plate of each finger/toe of both hands and feet was uneven, opaque, hypertrophic, and lacking in luster, with some grayish-black, punctate, grooved changes, and some of the distal nail margins were missing (Fig. 1). Oral mucosal involvement was not observed. The patient had a 5-year history of essential hypertension, which was well-controlled with long-term oral irbesartan, and a 2-year history of foot and nail ringworm, which was poorly controlled with oral and topical antifungal therapy. The patient had a history of smoking and alcohol consumption for >50 years, denied drug allergies, and had a family history of psoriasis. Before the onset of pemphigus, the patient had been treated with conventional oral antihistamines and topical glucocorticoid ointment, and had never received ultraviolet radiation.

**Figure 2.**
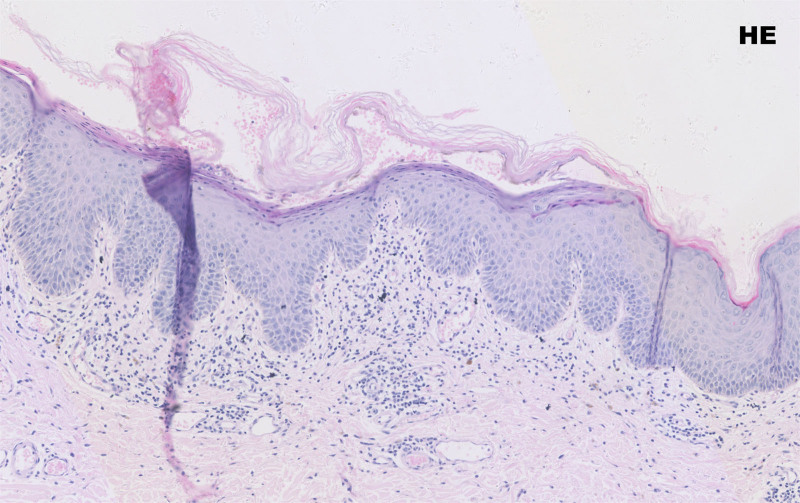
Hematoxylin and eosin (HE), original magnification 10×.

**Figure 3.**
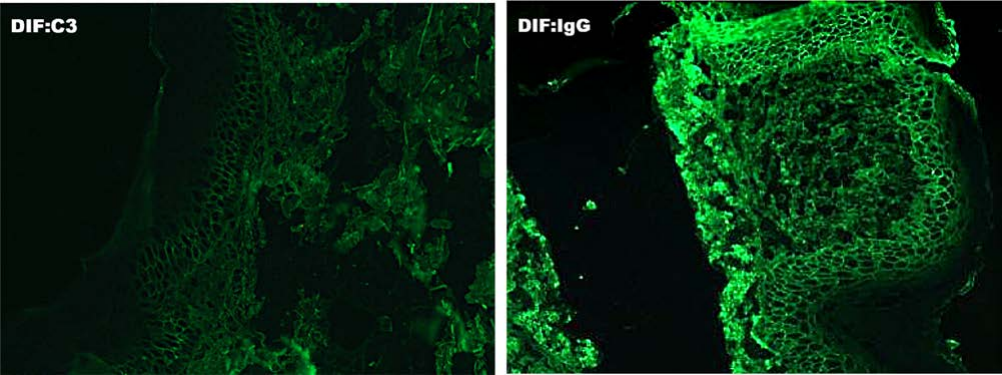
Direct immunofluorescence (DIF) revealed reticular deposition of C3 and IgG between subepidermal prickle cells.

Laboratory data revealed a uric acid level of 550.50 μmol/L. The desmoglein core protein 1 antibody was detected at 137.185 RU/mL (<20 RU/mL), the desmoglein core protein 3 antibody at 128.146 RU/mL (<20 RU/mL), and no IgG to BP180 or BP230. Blood tests, liver function tests, electrolytes, cardiac markers, infectious markers, tumor markers, tuberculin tests, and chest CT showed no significant abnormalities. Dermoscopic examination of the toenails showed changes consistent with those of the psoriatic nails. A biopsy specimen obtained on admission from the left axillary lesion showed mild hyperkeratosis and fused hyperkeratosis of the epidermis, a localized focus of subgranular acantholysis, mild spongiotic edema, vasodilatation of the superficial dermis, and congestion and infiltration of peritubular lymphocytes, histiocytes, and a few neutrophils (Fig. 2). Direct immunofluorescence (DIF) revealed reticular lattice deposition of C3 and IgG between subepidermal prickle cells, which were negative for IgA, IgM, and Fib (Fig. 3). Based on his history, clinical manifestations, and ancillary findings, he was definitively diagnosed with PV.

On admission, methylprednisolone (40 mg/d) was administered in combination with conventional antihistamines such as ebastine and topical glucocorticoids. After 9 days of treatment, the patient’s lesions gradually improved and the methylprednisolone dose was changed to 32 mg/d. On the 21st day of hospitalization, the patient was discharged with oral prednisone acetate (40 mg/d). When the patient was followed up until the 4th month, the dose of oral prednisone acetate was decreased to 7.5 mg/d, the psoriasis lesions recurred, especially on the lower limbs, with scaly erythema, white scales, pustules and severe pruritus, the patient was again hospitalized in the dermatology department for systemic treatment for 14 days, and the patient was discharged from the hospital after taking oral prednisone acetate 20 mg/d. On April 26, 2023, the patient returned to our dermatology outpatient clinic for follow-up, and considering that the patient’s psoriasis was poorly controlled and prone to relapse after stopping the medication, he decided to activate the biologic treatment after communicating with the patient, and after ruling out the contraindications, he added secukinumab (300 mg/4 wk) subcutaneous injection along with oral prednisone acetate 20 mg/d for the treatment of psoriasis and pemphigus. On July 19, 2023, he returned to our department for follow-up and was considered to have recurrence of pemphigus. Methotrexate (10 mg/wk) in combination with prednisone acetate (20 mg/d) and secukinumab (300 mg/4 wk) was added for the treatment of psoriasis and pemphigus. The patient visited the dermatology department of our hospital for follow-up on March 01, 2024, and there was no recurrence of the disease with no new rash. The current dose of methotrexate was reduced to 7.5 mg/wk, the dose of acid prednisone was reduced to 5 mg/d, and there was no adjustment in the dose or dosing interval of secukinumab. There were no psoriasis or pemphigus lesions on the skin of the entire body, and the fingernails and toenails of the hands and feet normalized (Fig. 1). The follow-up is ongoing.

## 3. Discussion and conclusion

Psoriasis is a common and easily relapsing chronic inflammatory disease that usually affects the skin, nail, and joints. The etiology and pathogenesis of the disease are not fully understood, and are thought to be mainly related to genetic, immunological, infectious, and environmental factors. Immunological and genetic studies have identified IL-17 and IL-23 as the key drivers in the pathogenesis of psoriasis.^[1]^ Pemphigus is a rare autoimmune, intraepidermal, and herpetic skin disease. Genetic, environmental, and pharmacological factors may be involved in the pathogenesis of this disease.^[2,3]^ The pathogenesis is based on the disruption of epidermal cell junctions caused by desmoglein core protein antibodies, leading to loosening of acantholysis and resulting in vesicles and bullae on the skin and mucous membranes.^[2]^ The average age of onset of pemphigus is 45 to 65 years, although the average age of onset in South Africa and northeastern China is lower at approximately 45 years.^[4,5]^ The prevalence of pemphigus patients is lower in the elderly, according to a survey, the global prevalence of PV is 2.83/10^6,[6]^ and the survey data in Northeast China shows that PV in the elderly accounts for about 12.67%.^[5]^

Comorbidities of psoriasis refer to the fact that, in addition to skin symptoms, patients often have other systemic diseases, such as articular damage, cardiovascular, metabolic, hepatic, renal, autoimmune, and psychiatric disorders.^[7]^ Nail fungal disease is the most common cutaneous comorbidity of psoriasis, with a prevalence ranging from 13% to 47%.^[8]^ In recent years, autoimmune herpetic diseases have been identified as new comorbidities of psoriasis, the most common of which is bullous pemphigoid,^[7]^ and the coexistence of psoriasis and pemphigus is relatively uncommon. A meta-analysis by Kridin^[9]^ showed an overall incident comorbidity rate of psoriasis in patients with pemphigus (2.4%; 95% CI: 1.0–4.4). In another study, patients with psoriasis were more than 3 times more likely to develop pemphigus than controls (HR: 3.25), and the risk was particularly high in women with psoriasis (risk ratio, 4.20).^[10]^ The available literature suggests an association between psoriasis and pemphigus, but this association requires further investigation.

The pathogenesis of the comorbidity in our patient is unknown, but the pathogenesis of psoriasis combined with pemphigus may be related to the hypothesis that the “epitope diffusion phenomenon,” i.e., the primary inflammatory process in psoriasis, may induce changes in epidermal proteins, thereby exposing certain antigenic epitopes previously hidden from the immune system, a process that may eventually induce a secondary autoimmune humoral response.^[11]^ Human leukocyte antigen (HLA) is a common genetic factor in psoriasis and pemphigus, with the HLA-DRB1 allele being associated with both pemphigus and psoriasis.^[12,13]^ Fibrinogen activator levels are elevated in psoriatic lesions, and fibrinogen activation is thought to be involved in the process of acantholytic release in pemphigus.^[14]^ The development of pemphigus may also be associated with anti-psoriasis treatments, especially ultraviolet (UV) radiation. UV light can cause epidermal instability and spiculation relaxation in the non-lesional areas of patients with pemphigus, which may exacerbate the triggering effect of UV light in phototherapy for psoriasis.^[15]^ However, this patient was not treated with UV, so the correlation between pemphigus production and UV treatment needs further investigation. T lymphocytes are key factors in the immune mechanism of psoriasis and IL-17A produced by Th17 cells acts on keratinocytes to promote endothelial cell proliferation. Eming^[16]^ suggested that patients with pemphigus produce IgG antibodies on the surface of keratin-forming cells, leading to the loss of intercellular adhesion, and that the production of such antibodies is dependent on T lymphocytes. Ye^[17]^ proposed that the levels of Th17-related cytokines such as IL-17 and IL-23 correlate with the severity of pemphigus disease. This overlaps with the T lymphocytes, which are key factors in the pathogenesis of psoriasis. In conclusion, the pathogenesis of psoriasis with pemphigus is complex, and requires further investigation.

Currently, no clear treatment options are available for psoriasis with pemphigus. As psoriasis and pemphigus share common disease targets, some researchers have found that *Tripterygium wilfordii* polyglycosides may be used to treat them through a potential co-therapeutic mechanism involving the inhibition of the IL-17 pathway and Th17 cell differentiation.^[18]^ Rallis^[19]^ reported the first successful treatment of coexisting plaque psoriasis and PV by using cyclosporine monotherapy. In addition, a few cases of successful treatment of psoriasis combined with pemphigus using rituximab,^[20]^ methylprednisolone in combination with methotrexate,^[21]^ and cyclosporine^[22]^ have been reported. In our case, the patient was treated with moderate-dose glucocorticoids combined with secukinumab and methotrexate, which improved both the psoriasis and pemphigus lesions. However, the pathogenesis of the combination of these 2 diseases remains unclear. Based on the common inflammatory pathway and the fact that secukinumab can block the Th17 pathway, it has been hypothesized that secukinumab, a biological agent for the treatment of psoriasis, may be effective in the treatment of PV. This patient had nail psoriasis, and damage to the nail was significantly better after 11 months of secukinumab treatment than before treatment, which is in line with the results reported in the available literature.^[23]^ We conclude that secukinumab is an effective treatment option for nail psoriasis. The patient’s current treatment regimen included secukinumab injections (300 mg/4 wk) and methotrexate tablets (7.5 mg/wk) in combination with prednisone acetate tablets (5 mg/d). We suggest adjusting the dose or the interval between doses for secukinumab injection after tapering off methotrexate tablets.

In this case, we believe that secukinumab was effective for the treatment of psoriasis vulgaris and PV, which may be related to their common inflammatory factors. In conclusion, the efficacy of moderate-dose glucocorticoids combined with secukinumab and methotrexate in the treatment of psoriasis vulgaris combined with PV and nail psoriasis warrants further investigation.

## Author contributions

**Funding acquisition:** Ran Wu.

**Resources:** Shaoqin Sun, Ran Wu.

**Writing – original draft:** Lanying Wang, Guixian Guo, Shanshan Tang.

**Writing – review & editing:** Ran Wu.
